# Research progress on human adenovirus sepsis

**DOI:** 10.3389/fped.2025.1552958

**Published:** 2025-05-19

**Authors:** Wei Jianhua, Zeng Lingjian, Huang Yanhao, Liao Jing, Liu Enmei, Zang Na

**Affiliations:** ^1^ Department of Respiratory, Children's Hospital of Chongqing Medical University, National Clinical Research Center for Child Health and Disorders, Ministry of Education Key Laboratory of Child Development and Disorders, Chongqing, China; ^2^ Department of Respiratory, Chongqing Key Laboratory of Child Rare Diseases in Infection and Immunity, Chongqing, China

**Keywords:** human adenovirus (HAdV), viral sepsis, pathogenesis, treatment, epidemiology

## Abstract

Human adenovirus is a significant viral pathogen causing lower respiratory tract infections in children, prone to developing into severe pneumonia and systemic inflammation with a high mortality rate, especially in immunocompromised children, drawing widespread attention worldwide. Sepsis, a life-threatening organ dysfunction caused by a dysregulated inflammatory response to infection, has historically been focused on bacterial origins. However, nearly all viruses can cause sepsis, which is often underestimated in clinical settings. In recent years, severe infections and even sepsis caused by adenovirus have shown a trend of periodic outbreaks. Early diagnosis of adenovirus-induced sepsis can not only prevent the overuse of broad-spectrum antibiotics but also ensure that patients receive timely and appropriate antiviral treatment. This article aims to provide a comprehensive review of the epidemiology, pathogenesis, diagnostic methods, and recent advances in treatment strategies for viral sepsis caused by adenovirus.

## 1 Introduction

Sepsis is a syndrome characterized by abnormal physiological and pathological reactions caused by infection (bacteria, viruses or other pathogens) and is one of the major causes of death in children (1, 2). There are many cases of viral sepsis in patients with negative blood cultures (3–6), However, in actual clinical diagnosis and treatment, these viral causes are often underestimated, resulting in an unnecessary increase in the use of broad-spectrum antibiotics and missed opportunities to use antiviral treatment plans, which increases the disease burden (7). Human adenovirus (HAdV) is an important viral pathogen that causes lower respiratory tract infections and severe pneumonia in children under 5 years old, accounting for 3.5%–11% of community-acquired pneumonia in children (8, 9), The severe infection rate is as high as 37% (10, 11), and the mortality rate can be as high as 50% (12), There are a large number of cases of viral sepsis caused by adenovirus in severe infections. Some studies have shown that early use of antiviral strategies and neutralizing antibodies can improve the prognosis of severe adenovirus infection (13–15), therefore, this article reviews the latest research progress in the definition, epidemiology, pathogenic mechanism, diagnosis and treatment of adenovirus sepsis, in order to provide a certain reference for early clinical decision-making for adenovirus sepsis.

### 1.1 Definition

In 2015, the third edition of the international consensus on sepsis defined sepsis as a life-threatening organ dysfunction caused by a dysregulated host response to infection. Unlike the first and second editions of the sepsis definition (16, 17), the third edition no longer uses the systemic inflammatory response syndrome (SIRS) to define sepsis, but it still plays an important role in the early identification of severe infection (18). Today, domestic and international research on the etiology of sepsis mainly focuses on bacterial infection. Studies have shown that about 1/3–2/3 of sepsis patients have negative blood cultures, among which viral sepsis cannot be excluded (3–6). However, there is currently no clear definition of viral sepsis. Since the latest definition of sepsis does not specifically limit the pathogen, viral sepsis is also applicable to this definition (1, 7). The most common site of infection in sepsis patients is the respiratory tract (64%–68%) (19). Severe infections caused by HAdV are often caused by lower respiratory tract infections (12), and there are manifestations of persistent disseminated viremia. The increase in serum viral load and persistent viremia are risk factors for severe prognosis and death in children (13, 20). Therefore, combined with the diagnostic basis of adenovirus infection, adenovirus sepsis is considered to be defined as cases in which adenovirus is confirmed as a clear pathogen through pathogen detection [antigen detection, adenovirus polymerase chain reaction (PCR) detection], adenoviremia exists, and the international consensus definition of sepsis is met. However, it is still very challenging to clarify the causal relationship between HAdV infection and sepsis, because a positive result of HAdV alone is not sufficient to diagnose adenovirus sepsis. It is also necessary to consider whether there is a concurrent infection and whether a secondary infection has occurred. Clinicians need to make a comprehensive judgment based on their clinical characteristics. The causal relationship between HAdV infection and sepsis needs to be further and better determined in other prospective cohort studies.

### 1.2 Epidemiology

**Figure 1:**
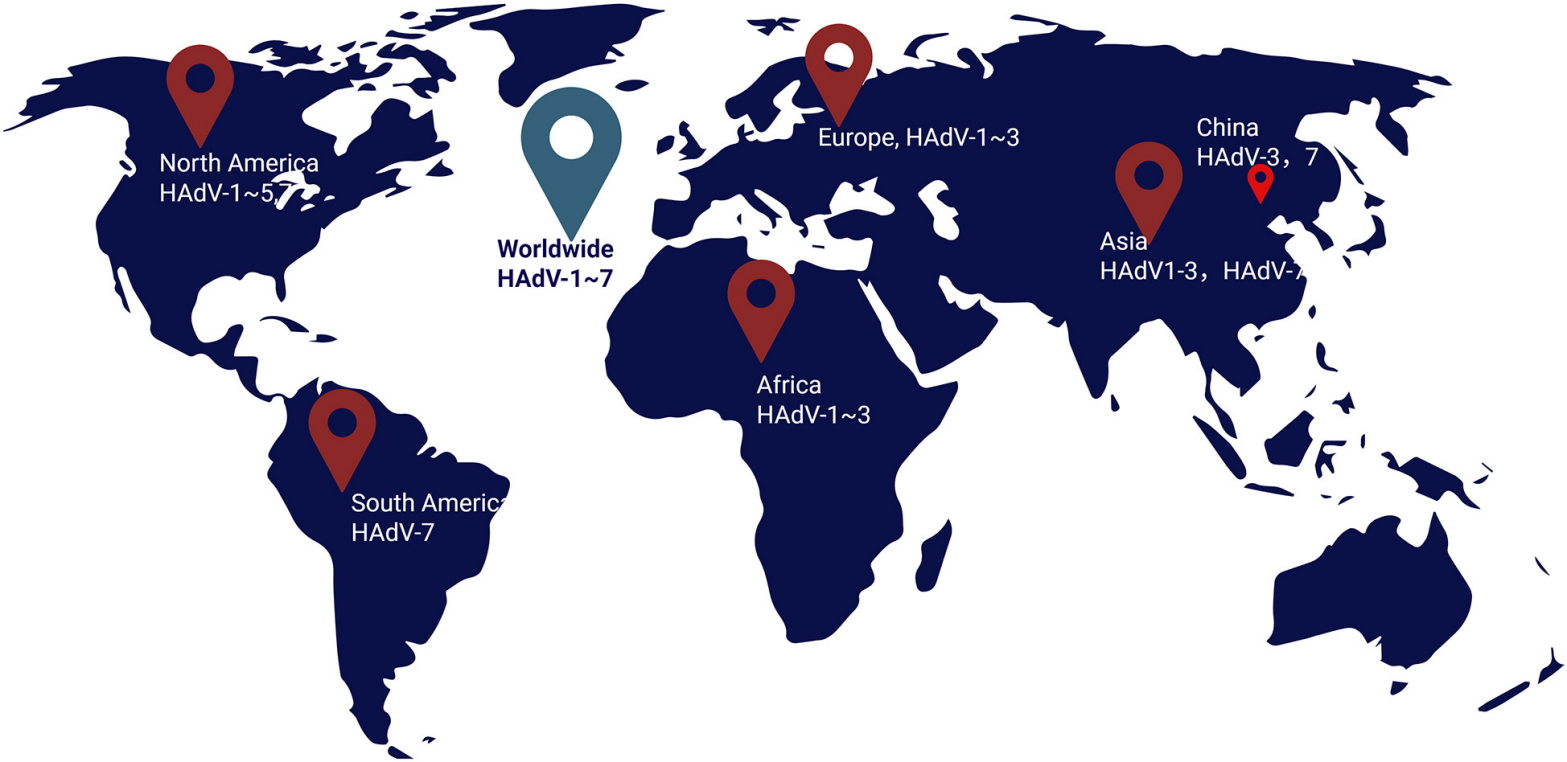
The worldwide map of molecular epidemiology trends of adenovirus (the predominant adenovirues genotype worldwide were marked).

Currently, the diagnosis of viral sepsis is often neglected, and the incidence of viral sepsis is often underestimated. However, a recent study included patients diagnosed with viral pneumonia and found through retrospective analysis that 61% of patients had viral sepsis, and HAdV is one of the main pathogens causing viral sepsis (21). According to different biological characteristics, HAdV is divided into seven subgenera from A to G. A total of 116 genotypes have been found, and they are still being updated (http://hadvwg.gmu.edu/). Different types of HAdV have different tissue tropisms. Their pathogenicity, epidemic trends and other characteristics are also different. HAdV infection in patients with normal immune function is usually self-limiting, such as infections of the eyes, upper respiratory tract, and gastrointestinal tract (12). However, HAdV is prone to cause lower respiratory tract infections in children (the proportion is 3.5%–11.0%) (8, 9), and is prone to develop into severe cases (the severe cases rate is about 37%) (10, 11). Severe patients may have respiratory failure, intracranial infection, and even death, including children with adenovirus-induced sepsis. Adenovirus types 3, 7, 14, 21, and 55 in the B subgenus, types 1, 2, 5, and 6 in the C subgenus, and type 4 in the E subgenus are commonly detected in respiratory infections in children (12, 22). In China, HAdV-3 (32.73%) and HAdV-7 (27.48%) are the main detected types (23). In Asia, HAdV-1–3 and HAdV-7 are the main detected types (24, 25). In Europe, HAdV-1–3 (45.6%) are the main detected types (26). In North America, represented by the United States, adenovirus type 3 (39.7%) is the most commonly detected type among civilians (including children and adults), and adenovirus types 1–5 and 7 are the main detected types (89.7%) (27, 28). In many countries in South America, HAdV-7 is the predominant strain associated with respiratory infections requiring hospitalization (29). In African countries, HAdV1- is the predominant adenovirus detection type (30, 31). Worldwide, HAdV-1–HAdV-7 are the most common types, while HAdV-4 and HAdV-5 have the highest serum positivity rates (32) (Figure 1). However, HAdV-7, HAdV-55, HAdV-4, and HAdV-14 often cause outbreaks in relatively closed environments such as military camps and schools (33). Types 7, 55, and 4 are more likely to be combined with severe pneumonia, leading to viral sepsis and endangering the lives of children (34–37). This is especially true for children under 2 years old, those after organ transplantation, and those with congenital immunodeficiency (38). In adults and children with normal immune function, there have also been reports of severe infections and even death caused by adenovirus (13, 39).

With the easy availability of rapid viral diagnostic testing, HAdV infections could be identified more often. After comprehensively analyzing relevant literature, Lin, G.L. et al. believed that disseminated disease and adenovirus sepsis in children account for 2% of all adenovirus infections, with a mortality rate of up to 55%. The main risk factors for the development of sepsis are immunosuppression (especially allogeneic hematopoietic stem cell transplantation), young children (less than 18 months old), and infection with highly virulent adenovirus serotypes (such as HAdV-7) (7). A recent retrospective cohort study found that among all patients hospitalized for viral pneumonia with sepsis, the detection rate of adenovirus was 8% of all viral sepsis caused by non-influenza viruses (21). A multicenter study on the risk factors for death from pediatric sepsis found that serum HAdV-DNA-positive adenovirus sepsis accounted for approximately 10% of viral sepsis, and after adjusting for factors such as age, child mortality risk score, previous health status, and immunocompromised status at the onset of sepsis, the adjusted odds ratio (AOR) of HAdV was as high as 3.50 (*P* = 0.006), indicating that adenovirus viremia is closely associated with increased mortality (40). In a retrospective study of 415 immunocompetent children hospitalized for adenovirus infection, a mortality rate of 15% was observed (41). In a recent cohort study of patients hospitalized for severe adenovirus pneumonia and transferred to the ICU, a mortality rate of 31.1% was observed (13). Among these deaths, a large number of cases met the diagnosis of adenovirus sepsis as described above. However, more detailed epidemiological data on adenovirus sepsis urgently need to be supported by more systematic multicenter epidemiological studies.

### 1.3 Pathogenic mechanism

**Figure 2:**
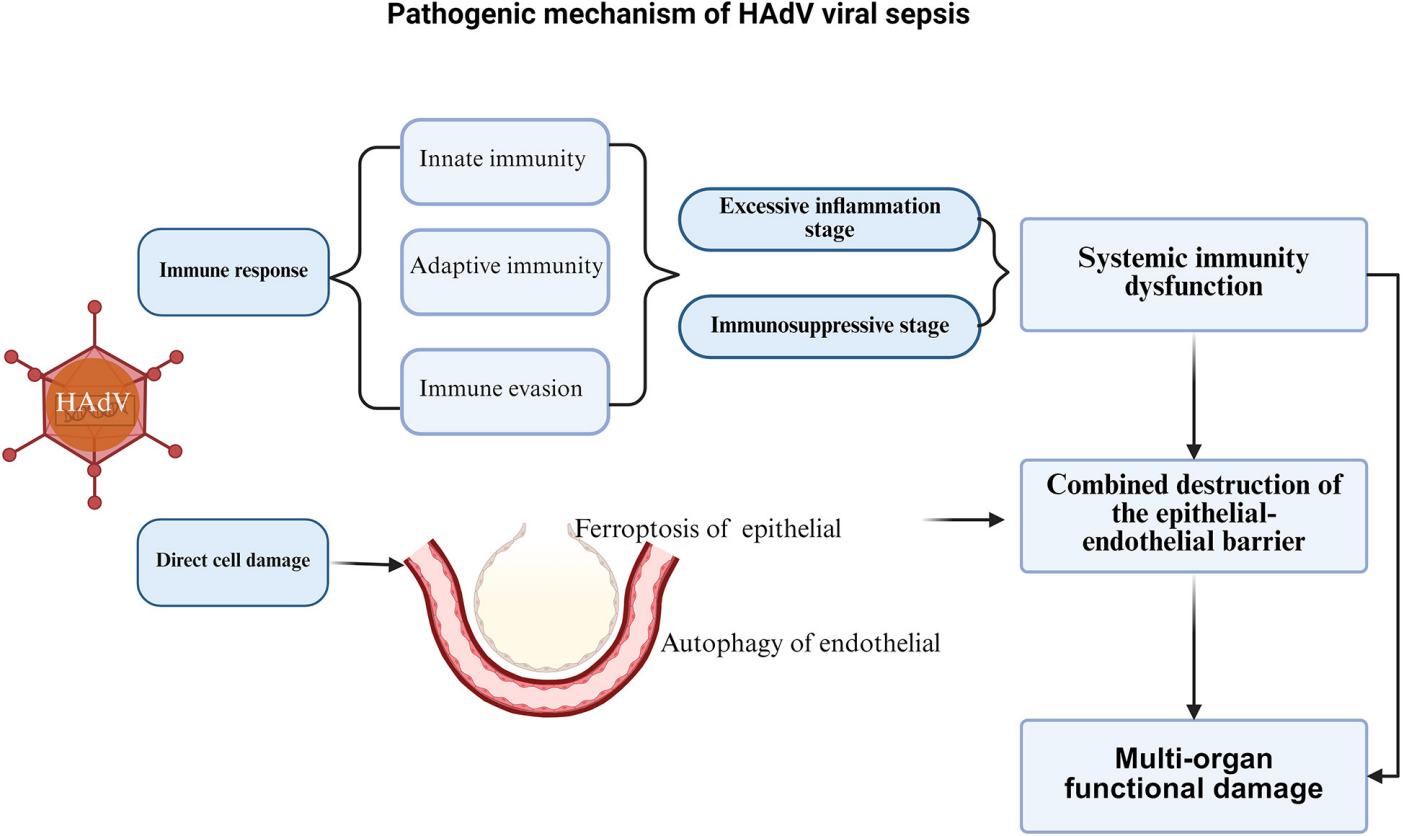
Pathogenic mechanism of adenovirus sepsis.

The pathogenesis of HAdV viral sepsis is limited, but similar to bacterial sepsis, its core is life-threatening organ dysfunction caused by the dysregulated host response to infection, which mainly involves systemic immune dysfunction, combined destruction of the epithelial-endothelial barrier, and a series of multi-organ functional damage caused by infection, including the respiratory system, cardiovascular system, nervous system, blood system, endocrine system, etc. (19) (Figure 2).

#### 1.3.1 Systemic immune dysfunction

HAdV is a non-enveloped icosahedral double-stranded DNA virus with a viral capsid protein surrounding the viral core. The virus core is composed of genomic DNA and core proteins pVII, pV, Mu (pX), pIVa2, terminal protein (TP) and protease encoded by AdV. The adenovirus core protein plays an important role in degranulation, genome nuclear transfer, progeny virus encapsulation and release (42). Capsid protein is divided into major capsid protein and minor capsid protein. The major capsid protein is composed of hexon, penton base and fiber, and the minor capsid protein is composed of pIX, pIIIa, pVI and pVIII. These proteins play a very important role in stabilizing the protein-protein interaction structure of virus particles (42, 43).

The immune dysfunction of HAdV viral sepsis is mainly manifested in the inability of the host's innate and adaptive immune response to HAdV to restore homeostasis, ultimately leading to a pathological syndrome characterized by persistent excessive inflammation and immunosuppression (44).

##### 1.3.1.1 Innate immunity

Like influenza virus and new coronavirus, HAdV first activates innate immunity after infecting the host. This initial host perception of pathogens is mediated by pattern recognition receptors (PRRs), including toll-like receptors (TLRs), retinoic acid-induced gene-1-like receptors (RLRs), nod-like receptors (NLRs) and c-type lectin receptors (45, 46). After HAdV binds to adhesion receptors through the fiber knob, it mediates endocytosis and signaling cascades through the binding of Penton to integrin receptors (such as αvβ5), and then the virus degranulates to the cytoplasm and transports to the vicinity of the nuclear pore. After further degranulation, the genetic material is transported to the nucleus (47), and pathogen-associated molecular patterns (PAMPs) are expressed. PRRs activate innate immunity by recognizing PAMPs. Normally, the innate immune system clears viruses by promoting the release of cytokines and chemokines (tumor necrosis factor, TNF, interleukin, IL-1β, IL-12 and IL-18), the recruitment of phagocytes, and the local activation of the complement and coagulation systems (44, 46). For example, Penton binding to integrins can activate phosphatidylinositol-3-hydroxykinase (PI3K) signaling, which can increase viral intracellular transport and trigger the production of the proinflammatory cytokine TNFα (48, 49). TLR9 can detect adenovirus in the cytoplasm, conduct signal transduction through the MYD88-dependent pathway, activate transcription factor NF-κB and other pathways, leading to the production of proinflammatory cytokines IL-6 and IL-8, and the release of IFNα/β (47, 50, 51). Activation of the NF-κB pathway can also promote the maturation of IL-1β and IL-18 and the release of necrosis factor and proinflammatory factor HMGB1 by inducing NOD-like receptor thermoprotein domain protein 3 (NLRP3) inflammasome activation (47, 52). In addition, adenovirus binding to membrane auxiliary factor CD46 can also induce complement activation, thereby inhibiting the release of adenovirus fiber and the exposure of protein VI (53).

##### 1.3.1.2 Adaptive immunity

The major capsid proteins of adenovirus (hexon, penton, and fiber) are highly immunogenic and can stimulate B cells to produce specific IgG antibodies, with the hexon protein being the primary target of neutralizing antibodies (54, 55). HAdV infection can also activate a CD4^+^ T cell immune response. T cell activation depends on MHC molecules presenting viral peptides: the MHC class I pathway degrades antigens via the proteasome to activate CD8^+^ T cells, while the MHC class II pathway degrades antigens via lysosomes to activate CD4^+^ T cells and promote B cell activation (56, 57). This T cell response exhibits cross-protective effects (56).

Autophagy degrades intracellular components (including pathogens) by forming autophagosomes and facilitates antigen presentation through the MHC class II pathway (58, 59). Additionally, the TRIM21 protein can bind to viral DNA-antibody complexes and degrade capsid proteins via the proteasome, thereby inhibiting viral replication (50, 60). Antibody-mediated endocytosis can also lead to viral uncoating, promoting viral clearance (60).

##### 1.3.1.3 Immune evasion

HAdV employs multiple immune evasion strategies to protect infected cells. The E3-encoded proteins disrupt MHC presentation, preventing CTL recognition (61). Specifically, E3-gp19K binds MHC I in the ER domain, blocking its transport to the cell surface and reducing CD8+ T cell and NK cell-mediated killing (62). Other E3 proteins (E3–14.7K, E3–10.4K, E3–14.5K, E3–6.7K) inhibit apoptosis by downregulating death receptors and suppressing NF-κB. E1B-19K and E1B-55K counteract p53-mediated apoptosis (63), while E1B-55K also suppresses IFN-induced gene expression and, with E4, inactivates the MRN complex to enhance viral replication (50). Early-expressed E1A blocks IFN-stimulated gene (ISG) transcription, further promoting viral persistence (64, 65). These mechanisms allow HAdV to evade immune clearance, sustain low-level viral release, and trigger chronic inflammation, contributing to long-term pulmonary complications.

##### 1.3.1.4 Excessive inflammation and immunosuppression

When the virus cannot be cleared by the host immune system, the homeostasis of the immune system will be broken, leading to excessive inflammation and immunosuppression, resulting in sepsis (66).

In the stage of excessive inflammation, HAdV viral sepsis is similar to sepsis caused by other reasons. It activates many PRRs through the release of injury-associated molecular patterns (IAMPs) by inflammatory cells such as leukocytes, macrophages, endothelial cells, and platelets. These PRRs in turn recognize PAMPs, further mediating tissue damage and leading to a vicious cycle (67, 68). At the same time, the excessive inflammation stage will also activate the coagulation system, complement system, neutrophils and vascular endothelium, causing organ damage and dysfunction (19, 69, 70).

In the immunosuppressive stage, innate immunity and adaptive immunity play a role simultaneously. With the increase of apoptosis of T cells, B cells and dendritic cells. T cells are exhausted, regulatory T cells and myeloid-derived suppressor cells (MDSC) are expanded (71, 72). In addition, in pathological conditions such as tumors, infectious diseases and autoimmune diseases, MDSCs can be rapidly accumulated and activated by IAMPs or PAMPs, and MDSCs are immature bone marrow cells that can hinder T cell function, thereby inhibiting immune response, inhibiting viral clearance, promoting viral persistence, and aggravating tissue damage (72).

#### 1.3.2 Combined destruction of the epithelial-endothelial barrier and multi-organ functional damage

The combined destruction of the epithelial-endothelial barrier caused by HAdV infection is also an important pathophysiological process for the occurrence of multi-organ function damage in HAdV sepsis (66). The inflammatory cytokine storm caused by persistent excessive inflammation in the early stage of infection is the main cause of endothelial dysfunction, loss of endothelial integrity, and multi-organ edema, leakage, and failure (19).

Besides, HAdVs infection can also directly damage host cells through various mechanisms. Different adenovirus serotypes enter cells by binding their fiber proteins to specific host cell surface receptors (e.g., CAR, CD46, DSG-2, etc.), triggering signal transduction and endocytosis. Following internalization, the virus undergoes uncoating - a process facilitated by adenovirus protease (AVP) that dissolves the viral protein capsid. The viral particles are then transported along microtubules to nuclear pores where the genome is released to initiate transcription. Subsequently, the virus penetrates the endosomal membrane and escapes into the cytosol, ultimately inducing cell death (73). When HAdV infects alveolar epithelial cells, inducing ferroptosis of alveolar epithelial cells, HAdVs directly damages the alveolar epithelial barrier, releases inflammatory mediators such as IL-6, IL-8, IL-18, and TNF-α, and mediates the increase of reactive oxygen species (ROS) and lipid peroxides (74). These inflammatory mediators and ROS can damage the endothelial cell. The endothelial cell damage can also be led by direct infection of HAdV through autophagy (75). Endothelial cell damage lead to the damage of epithelial-endothelial barrier and the polysaccharide coating on the surface of endothelial cells, leading to the degradation of glycocalyx, cell rearrangement, destruction of tight junctions between cells, and increased endothelial cell apoptosis, thereby damaging endothelial cells and increasing endothelial permeability (19, 75, 76). The endothelial cell damage lead to increased secretion of angiopoietin 2, which competes with anti-angiopoietin 1 for binding to Tie2 receptors, thereby activating RhoA enzymes, causing skeleton rearrangement and increased capillary permeability, leading to a vicious cycle (77), further aggravating the destruction of the endothelial barrier and damage to multiple organ functions.

### 1.4 Clinical manifestations

Similar to bacterial sepsis, adenovirus sepsis lacks specific clinical manifestaions (1). HAdV exhibit distinct tissue tropisms depending on their serotypes. Different adenovirus types could cause diseases in various tissues and organs, including pharynx, digestive system, liver, heart, urinary system, central nervous system, the respiratory tract and hematopoietic system (78). HAdV frequently causes ocular infections, resulting in self-limiting conditions such as simple follicular conjunctivitis and pharyngoconjunctival fever (79). HAdV is an important pathogen causing gastroenteritis and diarrhea in infants and young children. According to China's diarrheal syndrome pathogen surveillance data from 2009 to 2018, the detection rate of HAdV reached 9.27% (80). Recent evidence highlights HAdV's emerging role in severe pediatric hepatitis of unknown origin. While traditionally linked to immunocompromised hosts (e.g., transplant recipients), HAdV-41—typically causing gastroenteritis—has been implicated in unexplained hepatitis outbreaks, including cases requiring liver transplantation in immunocompetent children (UK, US) (81, 82). These findings challenge conventional paradigms and urge inclusion of HAdV in differential diagnoses for unexplained hepatitis, particularly in children. Besides, HAdV can induce myocarditis and dilated cardiomyopathy (DCM). In pediatric patients, these conditions may manifest with fulminant progression, severe cases can be life-threatening, necessitating early respiratory and circulatory support (83, 84). HAdV infections of the urinary system frequently cause hemorrhagic cystitis, predominantly occurring in school-aged children with immunocompromised status, particularly following renal transplantation or hematopoietic stem cell transplantation (85). In pediatric patients, HAdV-induced central nervous system disorders encompass febrile seizures, epileptic episodes, encephalitis, and meningoencephalitis. While mild cases typically demonstrate favorable prognoses, severe infections may progress to acute necrotizing encephalopathy, potentially resulting in permanent neurological sequelae (86).

HAdV is a significant viral pathogen of respiratory infections, predominantly affecting children aged 0–5 years and immunocompromised individuals. Notably, up to 20% of HAdV respiratory infections progress to HAdV pneumonia in neonates and infants (87). Adenovirus pneumonia often presents with persistent high fever, cough, and wheezing. Children with severe infection may experience persistent or difficult-to-correct hypoxemia and may also have extrapulmonary symptoms, such as poor mental and appetite, alarm, shock, and decreased blood pressure (13). There are a large number of cases of viral sepsis caused by adenovirus among patients with severe adenovirus pneumonia, which are manifested as acute respiratory distress syndrome (ARDS), persistent serum adenovirus dissemination, increased adenovirus load in serum and nasopharyngeal aspirate (NPA), systemic inflammatory cytokine storm, systemic immune cell exhaustion, and multiple organ dysfunction (13, 34, 35, 38, 88).

Notably, severe adenovirus (HAdV) spesis often leads to secondary hematological disorders, with hemophagocytic lymphohistiocytosis (HLH) being the most predominant (89). HLH is a rare, life-threatening immune dysregulation syndrome. While HAdV-associated HLH typically occurs post-hematopoietic stem cell transplantation due to immune dysfunction (90).

The condition manifests with persistent high fever, hepatosplenomegaly, cytopenia, coagulopathy, and hemophagocytosis on bone marrow smears. Due to insufficient understanding of its pathogenesis, mortality remains high [138–140]. Cases are distributed across all age groups—infants, older children, and adults—without significant predilection (89–91).

Early identification of adenovirus sepsis is of great significance for improving the prognosis of children. In recent years, there have been many reports on risk factors for severe illness or death caused by adenovirus infection. Among them, high serum viral load, highly pathogenic adenovirus serotypes (such as HAdV-7), combined immune deficiency, and young children are more recognized risk factors (13, 23, 34, 35, 38, 88, 92).

### 1.5 Treatment

#### 1.5.1 Pathogen-directed treatment of adenovirus sepsis

Sepsis guidelines emphasize the important role of early anti-infection treatment in improving the prognosis of sepsis patients and reducing mortality, and antiviral treatment should be initiated as early as possible for sepsis or septic shock caused by virus (1, 2, 92). At present, there are no prospective randomized clinical trials supporting the use of antiviral drugs for severe infections caused by HAdV, and no antiviral drugs have been approved by the FDA (93). Cidofovir is a viral DNA polymerase inhibitor and is the drug of choice for the treatment of adenovirus infection in immunocompromised populations. However, due to limited antiviral activity and nephrotoxicity, it is generally not recommended for use in immunocompetent patients (87, 94). Its potential therapeutic efficacy and safety in immunocompetent patients has only been confirmed in case reports and small sample group (95), more evidence is needed (15, 96). Compared with cidofovir, the lipid conjugate of cidofovir (Brincidofovir) has better bioavailability, no nephrotoxicity, and higher antiviral activity (97, 98). In an animal experiment, the anti-adenovirus efficacy of Brincidofovir was much higher than that of cidofovir (99). Another retrospective study on severe adenovirus infection found that Brincidofovir as a compensatory treatment could continuously reduce the viral load of 67% of patients and improve survival (100). However, the phase Ⅱ clinical trials revealed no statistically significant difference from the placebo treatment, and gastrointestinal toxicity has also been reported (101–103). The broad-spectrum antiviral drug ribavirin is a guanosine analogue with broad antiviral activity against RNA and DNA viruses. Studies have reported that ribavirin has a certain effect on severe adenovirus infection in immunodeficient children, newborns, and solid organ transplant patients (2/5), but its broad effectiveness and safety still need to be verified by prospective randomized controlled trials (104). Besides, Li et al. reviewed many other Nucleoside/nucleotide analogues (such as zalcitabine and alovudine), along with some natural compounds (such as cardamomin and phenolic compounds extracted from Camellia sinensis Kuntze), Epigenetic regulators inhibitors (such as valproic acid, vorinostat and trichostatin),and steroid-based compounds (such as digoxin, digitoxigenin and lanatoside C) to be potential candidates for HAdV therapy *in vitro* or *in vivo* (105) (Table 1).

**Table 1:** Treatment of adenovirus sepsis.

Therapy	Category	Compound/Reference	Type of study	Efficiency
Pathogen-directed therapy	Nucleoside/nucleotide analogues	Cidofovir (15, 87, 94–96)	Case report, retrospective study	Limited antiviral activity; nephrotoxicity
		Brincidofovir (97–103)	Retrospective study, basic research phase Ⅱ clinical trials	Better Bioavailability, and higher antiviral activity than cidofovir, no nephrotoxicity Phase Ⅱ clinical trials revealed no statistically significant difference from the placebo, gastrointestinal toxicity
		Ribavirin (104)	Retrospective study	Certain effect on severe adenovirus infection in immunodeficient children, safety uncertain
		Other (such as zalcitabine and alovudine) (105)	Review	Potential candidates for HAdV therapy *in vitro* or *in vivo*
	Natural compounds	As cardamomin and phenolic compounds extracted from camellia sinensis kuntze (105)		
	Epigenetic regulators inhibitors	As valproic acid, vorinostat, trichostatin (105)		
	Steroid-based compounds	Digoxin, digitoxigenin and lanatoside C (105)		
Immunomodulatory therapy	Glucocorticoids	(13, 92)	Prospective study, retrospective study	Controversy about the efficacy and timing of glucocorticoid use
	Intravenous immunoglobulin	(13, 93)	Prospective study, retrospective study	Improve the prognosis of severe HAdV infection, reduce the mortality
	Neutralizing monoclonal antibodies	(106)	Basic study	
	Highly effective antibody plasma	(14, 107)	Prospective cohort study, retrospective study	Improve the survival rate of patients, and no serious adverse reactions were reported
	Immunostimulatory therapy such as immune stimulators	Programmed cell death 1 (Pd 1)/Programmed death protein ligand 1 inhibitor, Ifn γ, Il 15, Il 7 (108, 109)	Review	Promote rapid clearance of pathogens, reducing the incidence of secondary infections and the mortality rate of late sepsis
	Adaptive immunotherapy	Adenovirus-specific t cell therapy (110, 111)	Case reports, multicenter study, clinical trial	Antiviral immunity, promote clearance of viremia, and reduce mortality
Supportive treatment/Complication management	Early fluid resuscitation and continuous assessment of hemodynamic stability and fluid responsiveness	(1)	International consensus	Reduce mortality
	Early respiratory support	Non-invasive or invasive mechanical ventilation, extracorporeal membrane oxygenation (Ecmo) (13, 28, 112–114)	Case reports, multicenter study, clinical trial	
	Treatment to enhance endothelial and epithelial barrier function	(19)	Review	
	Vasopressor therapy for septic shock, blood transfusion therapy for anemia, sedation and blood sugar control, nutritional support	(115)	Review	

#### 1.5.2 Immunomodulatory therapy

In the hyperinflammatory response stage of sepsis, glucocorticoids play a very important role in reducing the inflammatory response. However, there is controversy about the efficacy and timing of glucocorticoid use in severe infection. Some studies have pointed out that the use of glucocorticoids may be a risk factor for poor prognosis of severe HAdV infection (92), and some studies have pointed out that the timing and dose of glucocorticoid use have no effect on the mortality outcome of severe HAdV infection (13). Some studies have reported that intravenous immunoglobulin may help improve the prognosis of severe HAdV infection, especially in patients with hypogammaglobulinemia (93). For children with normal immune function, the early use of immunoglobulin may reduce the mortality outcome of children (13). The therapeutic effect of neutralizing monoclonal antibodies against HAdV fiber and penton on HAdV infection has been confirmed in animal models (106). In addition, a study involving 92 children with severe adenovirus pneumonia showed that highly effective antibody plasma screened from fresh frozen plasma of healthy blood donors can shorten the fever time of children with severe adenovirus pneumonia and improve the survival rate of patients, and no serious adverse reactions were reported (107). Such results were further confirmed in a prospective cohort study involving 59 children with fatal HAdV pneumonia (14). However, the therapeutic effect of monoclonal antibodies in humans still needs to be confirmed by more large-sample prospective cohort studies.

In the immunosuppressive stage of sepsis, immunostimulatory therapy can help restore immune function and promote rapid clearance of pathogens, thereby reducing the incidence of secondary infections and the mortality rate of late sepsis (108). Many immune stimulators have entered the animal experiment or clinical trial stage, such as programmed cell death 1 (PD 1)/programmed death protein ligand 1 inhibitor, IFN γ, IL 15, IL 7, etc. The clinical application prospects of these immune stimulators in adenovirus sepsis are worth looking forward to (109).

Adaptive immunotherapy such as adenovirus-specific T cell therapy may become a promising immunotherapy for severe HAdV infection. It has been reported that adaptive immunotherapy can be used to treat fatal adenovirus infection in hematopoietic stem cell transplantation (HSCT) patients with T cell exhaustion (110). A clinical trial of HSCT patients found that patients receiving Hexon adaptive T cell transplantation (ACT) had antiviral immunity for up to 6 months, viremia clearance rate of 86%, antigen-specific T cell response, and mortality rate decreased by about 90% (111) (Table 1).

#### 1.5.3 Supportive treatment and complication management

Supportive treatment for adenovirus sepsis is the same as for other pathogens, emphasizing early fluid resuscitation and continuous assessment of hemodynamic stability and fluid responsiveness. Notably, since adenoviral sepsis frequently develops from severe pneumonia caused by adenovirus infection, early respiratory support is critical for managing adenovirus sepsis. A 22-year U.S. study of hospitalized children with adenovirus infections revealed that 11.9% required non-invasive or invasive mechanical ventilation during hospitalization, while 0.4% underwent t extracorporeal membrane oxygenation (ECMO) (28). Recent researches indicates that the mechanical ventilation and ECMO might be beneficial for the patients with severe adenovirus infection (13, 28, 112–114). Besides, multiorgan failure is the one of the main causes of death in sepsis, so treatment to enhance endothelial and epithelial barrier function also plays a pivotal role in the treatment of adenovirus sepsis (19). In addition, vasopressor therapy for septic shock, blood transfusion therapy for anemia, sedation and blood sugar control, nutritional support, etc., all play a very important role in the treatment of adenovirus sepsis (115) (Table 1).

### 1.6 Discussion

Adenovirus is the main viral pathogen causing severe pneumonia in children, with a high mortality rate. It can cause adenovirus viremia. However, the incidence of viral viremia is often underestimated. Understanding the definition, epidemiology, pathogenic mechanism, diagnosis and treatment of adenovirus sepsis can provide a reference for early clinical decision-making of adenovirus sepsis, in order to improve prognosis and reduce disease mortality.

Brincidofovir is expected to become an effective anti-adenovirus treatment drug, and the early use of intravenous immunoglobulin may help improve the prognosis of severe HAdV infection. The application of many immune stimulatory factors and adaptive T cell transplantation therapy provide new therapeutic prospects for immunotherapy of severe HAdV infection.
